# Medical History of the Expedition to the Niger, during the Years 1841-2. Comprising an Account of the Fever Which Led to Its Abrupt Termination

**Published:** 1843-10-01

**Authors:** 


Medical History of the Expedition to the Niger, during the Years 1841-2. Comprising an Account of the Fever which led to its abrupt termination. By *James Ormiston M'William*, M.D. Surgeon of the Albert, &c. 8vo. pp. 287. Churchill, 1843.

Who has not heard of the ill-fated expedition to the Niger? It is curious that deaths and disasters, on a small scale, make a more vivid and distinct impression on our minds, and excite more acutely our sympathies, than wholesale slaughters and the destruction of whole fleets and armies. The shipwreck and sufferings of Byron and his little crew will be read and remembered, when the battle of Borrodino—perhaps even of Waterloo, will be forgotten! We can form a distinct idea of the personal misfortunes, privations, and hardships of an individual, or of a boats' crew—but the destruction of Napoleon's army, in the retreat from Moscow, overwhelms us with astonishment, and leaves no accurate picture on the mind's eye. On the other hand, we can easily conceive a dozen men groaning in fever, under a tropical sun, and between the decks of a small vessel, while one solitary man is left for duty—and he steering the vessel and attending to the engine without the assistance of a single hand!

The medical portion of the volume—the account of the fever—is a very small one—and we dare not indulge in giving much of the mere nautical narrative, which, though interesting to the public at large, is not adapted for a medical journal.

Every one knows that the extinction of the slave-trade was the benevolent purpose for which this unfortunate expedition was projected, besides the still greater object—the spread of Christianity among the natives of Africa. Three steam-vessels, the Albert, the Wilberforce, and Soudan, were fitted out with every possible attention to the health, the comfort, we had almost said, the luxury of the officers and crews. Divines, Medici, Geologists, Mineralogists, Botanists, Astronomers, &c. were liberally supplied—as well as a profusion of medical stores and culinary preparations. The crews of the three vessels consisted of 178 men, of which there were 53 officers, inclusive of civilians and engineers—67 white, and 28 coloured seamen. We must pass over the voyage outwards, as it does not present anything particularly interesting. The expedition entered the Mouth of the Niger on the 13th of August 1841, and two days afterwards, a German died of fever, with delirium and tremors, but not supposed to be of an endemic character. A considerable part of the narrative is occupied with the nautical difficulties attendant on the ascent of the river, and with descriptive sketches of the topography and population on both banks of that pestilential stream. These we must pass over. They had passed the great Delta of the Niger, and were preparing to explore the valley of that river, without any symptoms of illness, when, on the 4th of September (1841), a fever of the most malignant nature broke out in the Albert, and almost simultaneously in the other vessels—not abating till the whole expedition was entirely paralyzed! Between the 12th and 17th of September, the sickness still increasing, the model farm was established at Stirling Hill, and it was now evident that some decisive step must be taken. One vessel, therefore, the Soudan, was selected to descend to the open sea, and on to Fernando Po, with all the sick. She did so on the 19th September—but it was soon found necessary to despatch the Wilberforce in the same direction, leaving the Albert to prosecute the voyage farther up the river.

The settlement of the now famous MODEL FARM, at the confluence of the Niger and Tchadda rivers, includes a tract of land 16 miles by 5 in extent. Stirling Hill is two hundred feet above the level of the river, and near it rises Mount PATTEH, to the height of 1160 feet.

Having located the settlers, the Albert, sadly reduced in the number of her officers and men, but full of hope, started on her ascent of the river, with the view of reaching Rabba; but on the third day, (22nd Sept.) three officers, including the captain, were laid up with the fever—and three others, in the course of the evening, were added to the list. By the 3rd of October, there were only one white seaman, one sergeant and one private marine — Dr. Stanger, Mr. Willie, Mr. Huxley, and Dr. M'William, himself, capable of doing duty!! The season was advancing, the river beginning to fall, and, under such circumstances, it would have been sheer madness to attempt a further prosecution of the expedition. On the 4th of October, therefore, the Albert weighed, and began to drop down the fatal river. Dr. Stanger undertook to manage the engine, while our author was necessitated to work the ship, in addition to his professional duties. In steaming downwards, they touched at Stirling Hill, and found the white part of the new settlers in a sad plight. They therefore re-embarked all but the blacks, and continued their course towards the long-wished ocean. On the 16th October they fell in with the Soudan, at the entrance of the river, and both vessels proceeded to sea, bound for Fernando Po.

From this time, the expedition may have been considered at an end, though many of the officers and men fell victims after their departure from the pestiferous stream.

### VITAL STATISTICS.

Of the total number of whites in the squadron, viz. 145, there were no less than 130 cases of fever, and 40 deaths! There were 158 blacks, of which none died, though eleven had fever. This speaks for itself.

### The Fever.

There was little uniformity in the premonitory symptoms. A number of the patients had head-aches—scintillations along the spine—and chilliness, preceding the attack—others complained of debility, and some of burning sensation in the epigastrium. Then came giddiness, lassitude, tremor, foul tongue, small quick pulse, and general oppression. The eyes looked heavy—the pupils sluggish.

"*Character, course and duration of the symptoms.*—The accession was seldom accompanied by very marked shivering, yet previous to the period of vascular excitement, the patient usually experienced a sensation of coldness, and for the sake of warmth would fain have exposed himself to the rays of the sun. He would shortly express a wish to lie down, and would complain somewhat suddenly of increase of headach or giddiness, and intense heat of the skin, which had a dry parched feel, restlessness, intolerable nausea, and difficult breathing. The dyspnea in several instances, particularly in my own case, was extremely distressing, and continued from one to four hours, until relieved by spontaneous vomiting, or the occurrence of diaphoresis. Headach was with some the prominent symptom during the hot stage, and the feeling was described as that of a cord being tightly girded round the temples. The thirst was very urgent: the tongue was foul in the centre, moist, clean or reddish, and invariably marked by indentations on the edges. The countenance was more or less flushed, the eye occasionally suffused and always looked wild. Pulse rapid but small, frequently feeble; thirst urgent, bowels constipated, and urine passed often and in small quantity. There was in general tenderness of the epigastrium, sometimes acute, but often not discoverable unless upon pressure.

"In some cases, coldness of the stomach was complained of some days before death. A subsidence of febrile action in general followed in from three to six hours, or at all events, the symptoms if continued beyond the latter period became much mitigated. Diaphoresis came on, the thirst moderated, and the signs of oppression in a great measure disappeared. The principal complaint at this period was from *the disagreeable odour of the perspiration, particularly in those cases that subsequently proved fatal.* I was not sensible of this peculiarity in the smell of the perspiration in my own case, but I perceived it very distinctly in several others. The sweating continued until from eight to twelve hours had been occupied by the whole paroxysm. The patient, although considerably exhausted, expressed himself as free from all trouble, and the countenance also indicated improvement. This seemingly favourable change did not last long, for the accession generally returned in from six to ten or twelve hours. Occasionally the respite extended to twenty-four hours. In a few cases, there was a treacherous interval of forty-eight hours, in the early period of the disease; but these invariably assumed afterwards a low malignant type. The fever in them seemed to have rested only to give strength for a fresh accession.

"The accessions did not seem to observe any law of periodicity. They came on, disappeared, and returned at all hours of the day and night. The evening, however, was a more common time of accession than any other; in which case, after the cold sensation had passed off, the paroxysm generally ran through its stages in the course of the night, and had suffered a considerable remission by the hour of breakfast (eight) the next morning.

"In a few instances the remissions were as complete as in the interval of ague. These were, however, only exceptions to the general rule, for total absence of fever was indeed of rare occurrence during the course of the disease." 134.

If no material improvement took place by the 8th or ninth day, the prognosis was gloomy—the fever then assuming a low asthenic form. Local pain was seldom complained of—and, in several cases, the patients never made any complaint at all!

In favourable terminations, the remissions became more distinct, and the intervals more marked and lengthened—the countenance assuming a more natural expression—the skin more moist—the thirst diminished—and the pulse softer. At this period, diarrhœa was by no means uncommon—and also a copious diuresis, with a desire for food. Of the contingent symptoms the most prominent were delirium, yellowness of skin, and convulsions of various parts of the body. In no case was there "*black vomit.*"

### PATHOLOGY.

The following were the appearances in eight cases examined on board the Albert.

"*Head.*—In two cases where the head was examined, softening was found in the corpus callosum and walls of the ventricles. In one case there was a small quantity of serous fluid in the base of the brain, and an unusual proportion in the ventricles. The dura mater was always sound. The pia mater in one case red and injected. No subarachnoid effusion was observed.

"*Thorax.*—The contents of the thorax were in nearly all cases healthy in appearance. Adhesions between the costal and pulmonary pleuræ were found in one instance, with tubercular deposits in the lungs in the state of induration. In another, a cartilaginous state of the tricuspid valves, with serous effusion in the left pleural sac.

"*Abdomen.*—The peritoneum and its processes, as well as the surface of the intestinal tube, had in general a bilious tinge.

"*The Stomach.*—In several cases the stomach contained from one to five ounces of yellowish-green fluid. The mucous coat was invariably softened, whether this fluid were present or not. In three cases livid patches were variously distributed over the inner surface of the stomach, becoming more distinct when the mucous tunic was scraped off, exhibiting stelliform nuclei in their centres. In two cases, the livid marks were arranged in the form of parallel streaks. These pathological appearances were chiefly in the splenic extremity of the stomach and near the pylorus. In one case there was remarkable venous arborescence on the exterior of the stomach, attended with general engorgement of the portal system. Small points of ulceration were observed in three cases, and slight thickening of the mucous lining in one instance only.

"*Duodenum.*—The lesions observed in the duodenum were of the same nature as those in the stomach, but much less marked. In one case the lower portion of this gut contained a yellowish secretion, of the consistence of mucus.

"*The Jejunum* was free from disease, and likewise the ileum, until within three feet of its lower end, where were observed, softening of the mucous lining generally and livid spots. A series of small ulcerations were seen in four cases. In one, the membrane was thickened, rough, and the ulcerations had nearly perforated the bowel; this case proved fatal by terminating in dysentery. The agminated glands of Peyer were distinct and enlarged in three cases.

"*Colon.*—The colon was usually nearly empty. On these occasions a dark, bilious, pultaceous matter was found in this portion of the tube, but in small quantity only: it was viscid and tenacious, adhering to the mucous tunic: where lividity or ulcerated points were found at the lower end of the ileum, the same lesions were seen to exist on the arch of the colon. Softening of the mucous coat was remarkable in three cases. In that of the case of dysentery already mentioned, there was softening of the tunic where it was not ulcerated, and induration and elevation round the edges of the ulcerated patches.

"*Liver.*—The liver was congested in one instance; larger than usual in two cases. It was anemious in two cases where the patients died early, and on two other occasions when death took place long subsequent to febrile action. In the latter cases this organ was of a pale gray colour, and had a dry appearance on being sliced. This condition was not confined to one lobe.

"*Gall-bladder.*—The gall-bladder was distended with bile of the colour and consistence of tar in three cases: one of which was fatal on the third, one on the seventh, and the other on the ninth day. In another instance the gall-bladder was nearly filled with bloody bile. The man in this case died suddenly, many weeks after the fever had left him.

"The enlarged condition of Peyer's glands, which is regarded by Chomel and Louis as constant in the typhoid fever of France, occurred in three cases out of eight that were examined. In four cases, the subjects of which with one exception died early, slight ulcerations of the gastro-enteric mucous membrane were observed. This fact is worthy of attention, inasmuch as it would seem to imply that the cause of the river fever, in whichever way it is introduced into the system, induces an unhealthy action in mucous surfaces much more rapidly than even the low typhoid fevers of France. Chomel does not consider that ulcerations take place in typhoid fevers earlier than the twentieth day, when there is, also, softening of the mucous membrane around the follicles, or in that part of it which covers them. Louis found the patches of Peyer natural in twenty autopsies, made by himself, of yellow-fever cases at Gibraltar, during the epidemic of 1828.

"*Spleen.*—In one case the spleen was enlarged, soft, and breaking down under the fingers; in another enlarged, gorged with blood, but firm. This viscus was not altered from the normal condition in the other cases examined. The *pancreas* was not in any case otherwise than natural. The *kidneys* were mottled and larger than usual on one occasion. The *bladder* was in general collapsed. A case in which bloody urine was voided was not inspected.

"The morbid appearances observed in the intestines are very like those so often found in fatal cases of the typhoid fever of this country. This is not the place to recapitulate the evidence opposed to the doctrines of Broussais, regarding the nature of fever; but every day's experience tends to prove that the ulcerations and other lesions of the bowels are a specific effect of the fever poison, and not the cause of the fever itself." 147.

Morbid changes of this kind were found in the intestines *in all cases*—but none of these changes were constant—and therefore not essential to the fever. In two cases the blood was found in a fluid state after death.

The main sequence of the African fever was an irratibility of the mucous membrane of the bowels, continuing for a long time after the fever disappeared, and, no doubt, dependent on lesions contracted during the fever. This intestinal irritability occasionally rises into enteritis, and is then, too often, fatal.

*Hepatic disease* not unfrequently followed the gastro-enteric affection, either by sympathy, contiguity or extension.

### CAUSES.

The author has not been able to make any additions to what has long been known, or rather conjectured, respecting the etiology of remittent and intermittent fevers. That a certain something—the nature of which we know not—is emitted from marshy localities—is acknowledged by all, as causing miasmal fevers, &c—and that is the sum total of our knowledge!! The banks of the Niger offered no exception.

In respect to the latent period of the poison, our author remarks as follows:—

"When it is considered, as has been already noticed, that the vessels were constantly exposed to endemic influence, while they remained in the Niger, it is impossible to say at what time the miasmatous poison was first inhaled: but I hardly think that it was imbibed by any individual before we left the mouth of the river: if this were so, then fever may be said to have ensued on the sixteenth day* from the period of its earliest imbibition. Quarter-masters, seamen, and marines, whose duties were chiefly on deck, stokers in the engine-room, cooks, and in short, men of various occupations and constitutionally dissimilar, were simultaneously affected with fever.

"Upon the whole, I am inclined to think that in those cases which appeared at Iddah, the germs of the disease were contracted in the Delta. The stagnant state of the atmosphere, (relieved by occasional tornadoes,) and the causes of malaric exhalation being still abundant, and accumulated in the lower part of the atmosphere, from the want of a wholesome agitation, were favorable in its development at Iddah. Up to this point, the south-west breeze had always been felt during part of the twenty-four hours. The miasma in this state of condensation, so to speak, acted energetically upon men whose vital powers were already enfeebled, and who may have been for some time insensiby under its insidious influence.

"Moral causes came also into operation after leaving Iddah: many of those who were well were dispirited, and not a few, when taken ill, became speedily despondent.

"When out of the whole expedition there were only fifteen whites that were not attacked with fever in the Niger, it is scarcely possible to offer any opinion as to how far the susceptibility to this treacherous disease was influenced by temperament or idiosyncracy. Of the blacks, consisting of natives of various parts of Africa, including Kroomen, Americans, West Indians of African origin and East Indians, to the number of 158, eleven only were affected by the fever in the river: they (the eleven) had all been in England, and for some years absent from their respective countries. The disease in them assumed a comparatively mild form, and in no case did it prove fatal; showing that the immunity from endemic disease in warm countries, which is enjoyed by the dark races, is to a certain extent destroyed by a temporary residence in another climate.

"The question as to whether contagion contributed to the spread of the disease on board of the ships may, in my opinion, be briefly disposed of. All were exposed to the same influences; and nearly all were attacked with fever. Two only of the four medical officers who died had been in attendance on fever patients. Dr. Pritchett, Mr. Thomson, Mr. Stirling and Dr. Stanger were among the few who escaped being seized with fever, although they were in constant intercourse with the sick; and I was the last person in the Albert laid down with fever. The nurses on board the Albert were among the latest taken ill, and one escaped altogether. No fact came under my observation affording the slightest evidence that the disease was communicable from one person to another.

"Does one attack of river fever afford any protection against a second?

"My own experience, added to information obtained from many of my brother officers, and from Mr. King, the surgeon of the Ethiope, who has been more in

* "The Quorra, M'Gregor Laird's vessel, entered the river on the 19th of October, and fever broke out twenty-one days afterwards, on the 11th of November."

the Niger than any other medical man, is wholly unfavorable to the opinion that one attack of river fever affords any immunity from a second. On the contrary, those who have once suffered from this treacherous disease seem particularly predisposed to it, if they again venture within malarious influence." 181.

As a prophylactic, Dr. M'W. thinks himself authorised to recommend the daily use of quinine, with good diet, and a moderate allowance of wine.

### TREATMENT OF THE FEVER.

This is a very unsatisfactory subject. The very best mode of treatment is to get out of the range of the *cause* as soon as possible—that is to get out of the Niger into the open sea!—"Pessimum Ægro cœlum est, quod Ægrum facit."

*General bleeding* was not found to be successful in this fever. On the contrary, it was injurious. Local bleedings, as cupping the temples, were sometimes beneficial.

*Blisters* were often of great use.

*Mercury.*—In some of the Niger cases gentle ptyalism "certainly did good." In many cases "the full action of mercury *would* have been attended with danger." "Calomel combined with opium, and afterwards with quinine, appeared to me to be the best mode of exhibiting the remedy."

*Purgatives.*—The bowels were generally constipated, and required active purgatives, especially in the early stage.

"Calomel, jalap, and the bitartrate of potash were given at first, so as to cause free evacuations, which were in general dark or of a bilious character. After the bowels had been well emptied, castor oil and the milder aperients answered better than strong purges; which then indeed do much harm. Enemata were given with benefit, when epigastric tenderness or irritability of the stomach, rendered the administration of purgatives by the mouth inadmissible." 197.

*Diaphoretics.*—The best of these was the true James's powder, which kept up the action of the skin without producing nausea. Sometimes quinine was combined with it, and with good effect.

*Quinine.*—"In general when the tongue began to clean, and the other symptoms indicated that the functions were returning to their normal condition, quinine was given in large doses with great benefit. But it was not to this period alone that the use of this valuable remedy was restricted, for there were many cases in which, from the tendency to sinking from the very beginning, it was necessary to commence with quinine, wine, and light soups. In a disease like the Niger fever, so little amenable to treatment, no rule can be laid down for the exhibition of a particular remedy; but no medicine was found so efficacious as quinine in diminishing the severity of the paroxysms. In some of the more protracted cases, the red tinge over the sharp features would occasionally indicate that whatever power of reaction remained in the wasted system, was exerted to establish a feeble exacerbation, the exhaustion following which was often lessened by the liberal use of quinine.

"Brandy, wine, camphor, opium, and ammonia were freely given when the pulse began to flag, and when the symptoms generally denoted depression of vital energy; and often with almost miraculous effect, as in Case XIII.

"Sponging the body with tepid water and vinegar, in general afforded relief, but I never could carry the cold affusion further than the application of large wet clothes to the head. The warm bath was not much used, and in those cases in which it was tried the benefit obtained was only temporary, the relaxation and exhaustion produced by it, contra-indicated its general use in a disease which was marked by debility and tendency to sinking; tenderness at the epigastrium was, however, often relieved by applying to it a japanned case filled with hot water, and concave so as to fit closely to the abdomen. A large oblong case of similar construction was advantageously applied to the feet during the low stage of the disease; and at earlier periods, when the nervous depression retarded the development of the stage of re-action, in which case the extremities often continued cold after the chest and abdomen had become quite hot." 199.

The foregoing general remarks are illustrated by numerous cases with their details; but to these we need not refer. A good many pages are dedicated to the economy of the vessels, as ventilation, provisions, medicines, &c. &c. as well as to meteorology, &c. which will prove very interesting to naval surgeons, and naval officers whose destiny may lead them to the pestiferous shores of Africa—and especially into that fatal river, the Niger.

The volume is written with great modesty, and indicates, in every page, good sense, discernment, and both practical and scientific knowledge.

